# Sex-specific vertical movements of spawning atlantic cod in coastal habitats inferred from acoustic telemetry

**DOI:** 10.1038/s41598-024-74896-2

**Published:** 2024-10-06

**Authors:** J. E. Skjæraasen, E. M. Olsen, K. McQueen, D. Nyqvist, J. J. Meager, Ø Karlsen, L. D. Sivle

**Affiliations:** 1 Institute of Marine Research, Nordnes, Bergen, 1870, 5817 PB Norway; 2 Institute of Marine Research, Flødevigen Marine Research Station, His, 4817 Norway; 3 Centre for Coastal Research, Department of Natural Sciences, University of Agder, Kristiansand, 4630 Norway; 4 Department of Environment, Land and Infrastructure Engineering, Politecnico di Torino, Torino, Italy; 5 Natural Resources, GHD, 3 South Sea Islander Way, Maroochydore, Qld, Queensland, 4558 Australia

**Keywords:** Behavioural ecology, Marine biology

## Abstract

Fish spawning location and behaviour can be challenging to detect, especially in deep water. Here we utilise two large acoustic telemetry datasets from western and mid Norway to study the vertical movement dimension of individual Atlantic cod *Gadus morhua* spawning behaviour in their natural habitats. The datasets comprise ~ 6 million depth detections from 644 sexed, mature cod, collected at seven different Norwegian coastal cod spawning grounds during 2017–2021. During the spawning period, females were typically found at shallower depths compared to males, while this pattern was much less pronounced or absent outside this period. Furthermore, we identify a hitherto undescribed sex-dependent vertical movement behaviour: periodic descents, where females made deep dives, putatively associated with egg release and spawning, approximately every third day. Females not showing this behaviour tended to occupy deeper positions in the water column than the “periodic descent” females and hence may approach and retreat from spawning males more horizontally. These observed sex-specific behaviours correspond well to the lek-like description of the cod mating system and with cod reproductive physiology, where females are known to mature and release batches of eggs at intervals of several days as part of a bet-hedging strategy.

**Supplementary Information:**

The online version contains supplementary material available at 10.1038/s41598-024-74896-2.

## Introduction

The Atlantic cod *Gadus morhua* is an iconic cold-water fish of high ecological, cultural and commercial importance, distributed throughout North Atlantic coastal and shelf habitats^1^. Cod has been fished for millennia^2^, with several stocks suffering precipitous declines and population collapses^3,4^. Cod fisheries often target spawning aggregations, prompting early investigations into the cod mating system and their spawning behaviour.

Laboratory studies of cod spawning behaviour have revealed both male-male aggression and male courtship of females, including the prominent production of drumming sounds by males^5–8^. Female cod are batch-spawners, releasing up 20 batches during a lengthy spawning period, with a portion of eggs matured and spawned approximately every 2–6 days^9,10^. This probably conforms to a bet-hedging strategy that will buffer against environmental unpredictability to boost female fitness^11,12^. A key component of cod mating behaviour is the “ventral mount”, or mating embrace, whereby the male is positioned underneath the female, clasping her with his enlarged pelvic fins inducing gamete release by both sexes^5,7,13^. This particular spawning behaviour has been observed in several experimental studies^5,7,14^and in the wild^15^.The cod mating system has been described as a lek, where males form aggregations that females visit for the purpose of fertilisation, and where males offer no other resource than gametes^16–18^. The vertical dimension (i.e., depth use), appears integral to both the mating system of cod^19,20^and the spawning act itself^21,22^. To date, most studies on cod mating and spawning behaviour used to interpret vertical dynamics have been conducted in comparatively small tanks featuring relatively small spawning aggregations, whereas field studies typically have been based on only a few local populations.

Acoustic telemetry is a useful tool for studying animal behaviour in aquatic environments, and has provided insights into the mating systems of a variety species^23,24^including cod^17,19,25–27^. Coastal cod in Norway typically use sheltered inshore locations such as fjords and bays for spawning^28^, involving regional residency and some level of spawning site fidelity^12^. This ecotype^29^of cod is therefore a promising candidate for telemetry studies on spawning behaviour, as the cost of deploying and maintaining receiver arrays limits the spatial extent of spawning habitat that can be effectively monitored. Indeed, telemetry studies undertaken to date suggest that depth-related behaviour plays an important role in spawning aggregations of coastal cod in southern^20^and western Norway^19,30^, with males overall maintaining deeper positions than females throughout the spawning period. The depth of spawning locations will vary among sites depending on the local seascape, but often the spawning cod are found near the seafloor at depths from about 30–100 m^19^, present study).

**Fig. 1:**
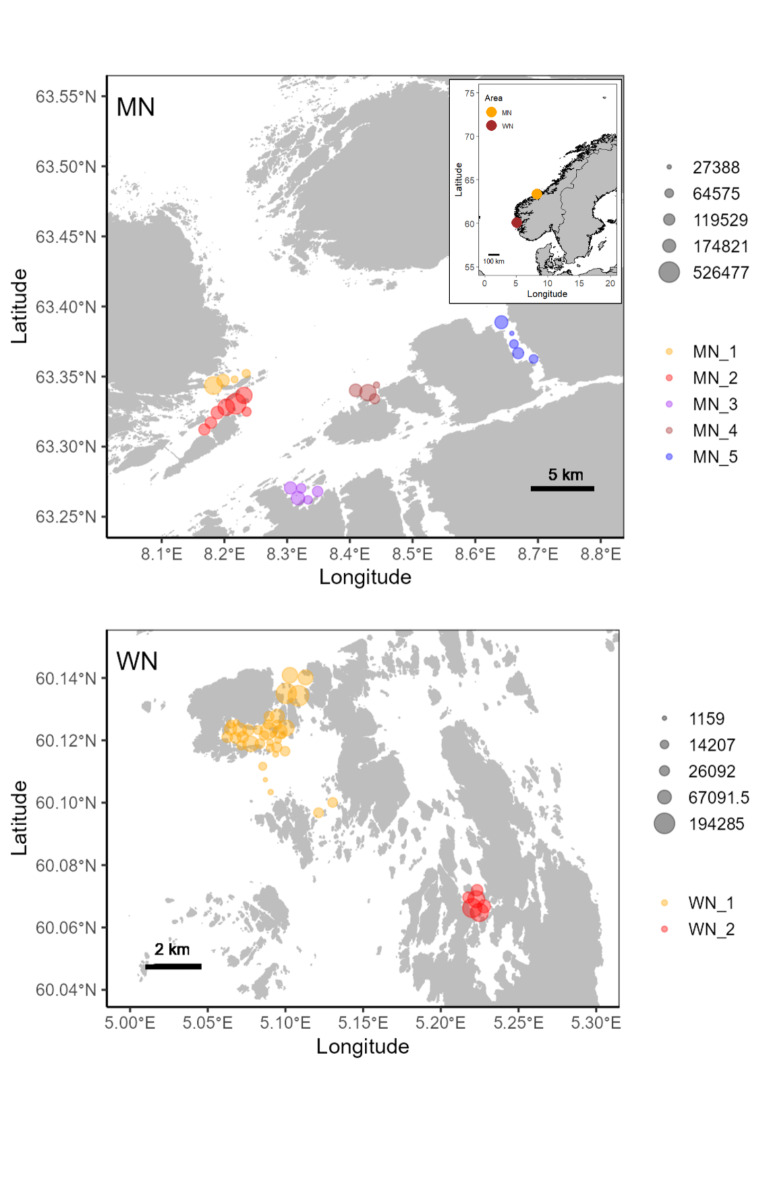
Locations of the study area. Insert, top panel, shows the two main locations on the Norwegian coast. Top panel, shows the location of the spawning grounds in mid Norway, botttom panel shows shows the location of the spawning grounds in western Norway. Different colours represent different spawning grounds. In the analyses all detections from stations (locations) having the same colour were treated as the same spawning ground. Size of points are scaled to the number of depth detections within panels. Map data were sourced from OpenStreetMap (https://www.openstreetmap.org/copyright).

Here we take advantage of two very large telemetry datasets on sexed, mature, and spawning cod collected in western (2019–2021) and mid Norway (2017–2019) (Fig. 1). Together, the datasets included in the present study comprise 6 million interannual depth detections from 644 sexed mature cod, obtained at seven inshore coastal cod spawning grounds monitored over multiple spawning periods. These large datasets provide a unique opportunity for a comprehensive investigation of the vertical dynamics of cod mating behaviour. We first test the prediction that the sexual segregation reported on local spawning grounds of western and southern Norway occurs more widely and is thus likely to be a general feature for Norwegian coastal cod. We then test the hypothesis that the vertical dynamics of cod behaviour will be sex-dependent since females are batch-spawners while males could reproduce more continuously throughout the spawning season^31,32^. Specifically, we predict that the batch-spawning strategy by females manifests in a periodic, depth-related signature in the telemetry data.

## Methods

### Study systems

We studied cod mating behaviour in two regions separated by about 450 km of coastline (Fig. 1). In western Norway (WN), we included two inshore spawning grounds, Bakkasund (WN_1) and Osen (WN_2) (Fig. 1). In mid Norway (MN), a total of five neighbouring spawning grounds were included: Glasøysvaet (MN_1), Lauvøysvaet (MN_2), Araneset (MN_3), Åkvika (MN_4) and Dromnessundet (MN_5) (Fig. 1). The two study regions had comparable maximum depths of about 80–100 m. For further details about the study systems, see McQueen et al^33^. and Skjæraasen et al^34^.

## Fish sampling and monitoring

Cod were caught during late January (WN) to mid-February (MN), i.e. just prior to or at the beginning of the spawning season, at each of the seven spawning grounds. Sampling in mid Norway was conducted during 2017–2019 while in western Norway sampling was conducted during 2019–2021. The fish were caught using baited pots (MN, WN), hook and line (MN), and gillnets (WN). The fish were kept in net pens within the study area post capture until tagging and release.

Prior to the tagging operation, fish were anesthetized in a bath of seawater and MS-222 (50 mg per l seawater) (WN), or a mixture of benzocaine (1.5 ml per 10 l seawater) and metomidate (5 ml per 10 l seawater) (MN). Subsequently, each cod was measured for weight and length, and sexed by ultrasound^35^. Egg biopsies were obtained from all females, except on the mid – Norway spawning grounds in 2017, and subsequent analysis of oocytes by image analysis allowed confirmation of sex and spawning readiness^36^. An acoustic tag was inserted in the body cavity through an incision made on the ventral side of the fish, which was then closed by two sutures. Each cod was tagged with one of four types of acoustic transmitter (V13P, V13TP, V13AP, V13TP ADST tags, Innovasea, Canada). These tags transmit unique identity codes (IDs) at 69 kHz and were additionally equipped with combinations of pressure (depth), acceleration or temperature sensors. Transmissions occurred at random intervals, on average every 250 s with a minimum and maximum delay of 200 and 300 s, respectively. Tags with more than one sensor alternated transmissions between the sensors. In the present study, only fish ID and depth information have been analysed, focussing specifically on the vertical aspect of movement behaviour. Additionally, an external T-bar tag (TBA standard anchor t-bar tag; Hallprint, Australia) was anchored at the base of the anterior dorsal fin for visual recognition of tagged fish. After tagging, cod were returned to a tank filled with a constant supply of seawater to recover from the tagging procedure, before being transported to central positions within their respective capture spawning grounds and released (Fig. 1). All methods were performed in accordance with the relevant guidelines and regulations and approved by the relevant authorities.

After release automated monitoring of individual cod presence and vertical movements was achieved by fixed arrays of acoustic telemetry receivers (VR2W and VR2Tx, Innovasea, Canada) deployed at all seven spawning grounds (Fig. 1). Receiver maintenance and data download was conducted at least once per year and usually during early summer (June), after the spawning season had ended.

Cod tagged at the WN_1 spawning ground were exposed to seismic airgun shooting for 5 days during the spawning periods of 2020 and 2021, as part of a behavioural response experiment. A minor, short-term increase in swimming depth was the only behavioural response to this exposure detected^33,37^. Given the low-level of the response, these data were retained in the dataset for investigating general spawning behaviours.

## Data preparations

Data were downloaded from the acoustic receivers and into a database (VUE Software, version 2.7.0, Innovasea) where fish detections were corrected for receiver clock drift using a linear correction based on the satellite clock time stamp at receiver initiation and download. The time-corrected datasets were exported as csv files and used for all further data analyses.

Pre - filtering the western and mid Norway dataset contained 9 225 394 and 10 630 290 depth detections from 204 and 547 cod, respectively. Prior to statistical analyses, the time-corrected data were filtered to (i) remove fish that were detected but did not exhibit vertical movement for > 1 day and thus were assumed to be dead^38^, (ii) remove duplicate data from fish tags detected at multiple receivers (only the first detection of the same fish was retained if the signal transmission were detected within 200 s at multiple stations) and (iii) dubious/erroneous detections, defined as a single daily detection of a given fish ID within the grid. This filtering process left 3 363 624 and 7 042 901 valid depth detections from 202 to 534 cod from western and mid Norway, respectively.

The present focus was on sexed mature cod only and their vertical behaviour during the spawning period and compared to behaviour outside of this period, with the latter hereafter defined as the feeding period. The spawning period was defined as February to March in western Norway^33^and February to April in mid Norway^34^. The transitional months between the spawning and feeding period, i.e. the months of January and April and January and May from the western and mid Norway datasets, respectively, were omitted from all analyses. Finally, to be able include fish length at tagging as a covariate and reduce the possibility of substantial individual differences in growth since sampling time, only data from the tagging year was considered. The final datasets used for analyses of overall depth contained 2 092 262 and 3 843 251 valid depth detections from 163 and 481 sexed mature cod from western and mid Norway, respectively (Table 1).

**Table 1:** Summary data for the present study. Area; the larger regional study area on the Norwegian coast for western (WN) and mid Norway (MN), the number in brackets refer to the number of spawning grounds monitored in each location. SP_M; the defined spawning period in months for each area. Study_Per; the present study period for the regional study areas. Fish were tagged and released every year at each spawning ground during the respective study periods. # cod; the number of mature cod retained in the final datasets post filterering and used for analyses of overall depth differences in the feeding and spawning period. Detections; the total number of detections in each dataset. #Codsp; the number of data – rich sexed mature cod selected for analyses, i.e. > 2000 detections and > 20 observational days, of periodic descents during the spawning period. Det_sp_dr; the total number of detections for the datarich cod in the spawning period. #Cfp; the number of data – rich cod selected for analyses of periodic descents during the feeding period, i.e. the months June to August. Det_fp_dr; the total number of detections for the data - rich cod in the feeding period.

Area	Sp_M	Study_Per	#cod	Det	#Csp	Det_sp_dr	#Cfp	#Det_fp
WN (2)	02–03	01/19 − 12/21	163	2 092 632	96	736 794	49	515 875
MN (5)	02–04	02/17 − 12/19	481	3 843 251	225	1 720 194	87	945 006

To study vertical movements associated with spawning, we explored the vertical movements of subsets of data-rich fish in more detail. As a conservative approach, only cod with at least 21 detection days and at least 2000 depth detections were considered as data - rich and included in these analyses. Analyses were undertaken on data from fish meeting these selection criteria during the spawning period and, for contrast, for data from western and mid Norway from the months of June to August to have a period (3 months) of similar length as the spawning periods, whilst avoiding the transitional months of April and May and maximising the number of fish available for analyses. In total 96, 44 females and 52 males, and 225, 93 females and 132 males, data - rich fish with a total of 736 794 and 1 720 194 detections were selected for analyses from the spawning period in western and mid Norway, respectively (Table 1). During the contrast feeding period 49, 26 females and 23 males, and 87, 39 females and 48 males, data - rich cod with 515 875 and 945 006 detections were selected for further analyses from western and mid Norway, respectively (Table 1).

## Statistical analyses

All data processing and analyses were undertaken in R (version 4.2.0, R Core Team 2021, https://www.r-project.org). Plotting was done with *ggplot2*(3.3.5)^39^, and mapping with *qqmap*(v3.0.0)^40^. Data were organized using the *tidyverse*(1.3.1) packages^41^. All model selection followed the same approach; an initial model was employed, followed by use of the ‘dredge’ command of the *MuMIn*package^42^ to arrive at the most parsimonious model with the lowest *AICc* score. All variables included in the best model or in a model within 2 *AICc* units of the best model were retained for the final model used in analyses^43^.

We first examined if there were consistent differences in vertical position between males and females whilst also examining the effect of fish body length and photoperiod (day or night) on fish depth use during the spawning- and feeding period at all the examined spawning grounds^33,34^. We employed the following initial model, run separately for (i) each spawning ground and (ii) the spawning and feeding periods, using the *lmer* function of the *lme4 package*^44^:

1 $$DEPTH = SEX* DAY\,NIGHT *LENGTH + FishID + Day\,of\,year/Year$$

The response variable DEPTH is the swimming depth of the fish corrected for tidal variation. SEX is the categorical explanatory variable of fish sex, DAY_NIGHT is a categorical explanatory variable having a value of zero if the depth recording occurred during the day and 1 if it occurred during the night, LENGTH is the continuous explanatory variable of fish length (cm) centred at the mean. *FishID* and *Day_of_Year* (Julian day) nested within *Year* were included as random effects. We used the *suncalc*package of R^45^ to obtain the daily time for sunset and sunrise in our study area. Day was defined as the time from sunrise to sunset and night conversely the time from sunset to sunrise. Tidal signals were removed from depth-sensor data by subtracting the difference between the current and average sea level from swimming depth to correct for tidal variation. Sea level data were downloaded from the Norwegian Mapping Authority, Hydrographic Service (https://www.kartverket.no/) using either a longitude of 5.149636 and a latitude of 60.080593 or a longitude of 8.343865 and a latitude of 63.382649, for WN and MN study areas, respectively. Sea level data are at 10 – minute intervals, and we used linear interpolations between each 10 min measurement point to get tidal data with a resolution of 1 s to match the resolution of the telemetry data before applying the correction.

To study vertical movements associated with spawning, we explored the vertical movements of the data - rich fish in more detail. Based on earlier studies (e.g^19^). we predicted that females would be closer to the surface than males during the spawning period, and, given the lek-like mating system of the cod, in combination with the batch - spawning strategy of females releasing batches every 2–6 days^9,10^, that they would periodically descend towards the males to mate and spawn. We therefore constructed an automated approach to detect this behaviour for the data – rich fish from the spawning period and, as a control, also used this automated approach for the data – rich fish from the control feeding period, i.e. the months of June - August. To identify what we hereafter term “periodic descents”, we used the following criteria: (i) the maximum depth recorded during a periodic descent day was at least 50% deeper than the average maximum depth from the day before and after calculated with a centred rolling mean, (ii) a maximum of 6 calendar days were allowed between descents in the same sequence, and (iii) at least two periodic descents were needed within a sequence for the cod to be denoted a “periodic descent cod”, given that females release multiple batches during a prolonged spawning period^9,10^.

To explore what affected the likelihood of a cod performing this behaviour, an initial model including the following explanatory variables (i) sex – categorical independent variable, (ii) length (cm) – continuous independent variable and (iii) their interaction was employed. Spawning ground, i.e. 7 levels, was included as a random effect. Periodic descent was treated as a binomial response variable with a value of 1 for cod that had performed the behaviour and 0 otherwise. The analyses were undertaken using the *glmer* function of the *lme4*package^44^.

In addition to our selected method for detecting the “periodic descent” behaviour described above we also tested eight different scenarios by relaxing or restricting our three selection criteria, i.e. (i) descent depth; either 50% or 100% deeper than the preceding and following day, (ii) time allowed between batches; either 6 or 10 days and (iii) the number or batches required within a sequence; either 2 or 3 batches. Summarised data from the scenarios tested is presented in the supplementary material (Supplementary Table 1).

Finally, we examined the time between female periodic descents during the spawning period, treating the time of maximum depth recorded during a periodic descent as a putative proxy for spawning time. To explore if the likelihood of nighttime versus daytime spawning differed between western and mid Norway we employed an initial model including region, i.e. western or mid Norway, treated as a categorical independent variable and FishID as a random effect. Photoperiod, i.e. day or night, was treated as a binomial response variable with a value of 1 for night and 0 for day in the model. The analysis was undertaken using the *glmer* function of the *lme4*package^44^ .

## Ethics and permits

Permits were given from the Norwegian Directorate of Fisheries to capture fish (permit 19/14024), and the Norwegian Petroleum Directorate gave permission to conduct seismic shooting (permit 739/2019). The experiment, including all animal sampling and tagging methods were approved by the Norwegian Authority for Animal Welfare (FOTS ID 8579, 16616, 18034 and 26019). Methods are reported in accordance with ARRIVE guidelines 2.0 (https://arriveguidelines.org/arrive-guidelines).

## Results

### Sex-specific depth use

**Fig. 2:**
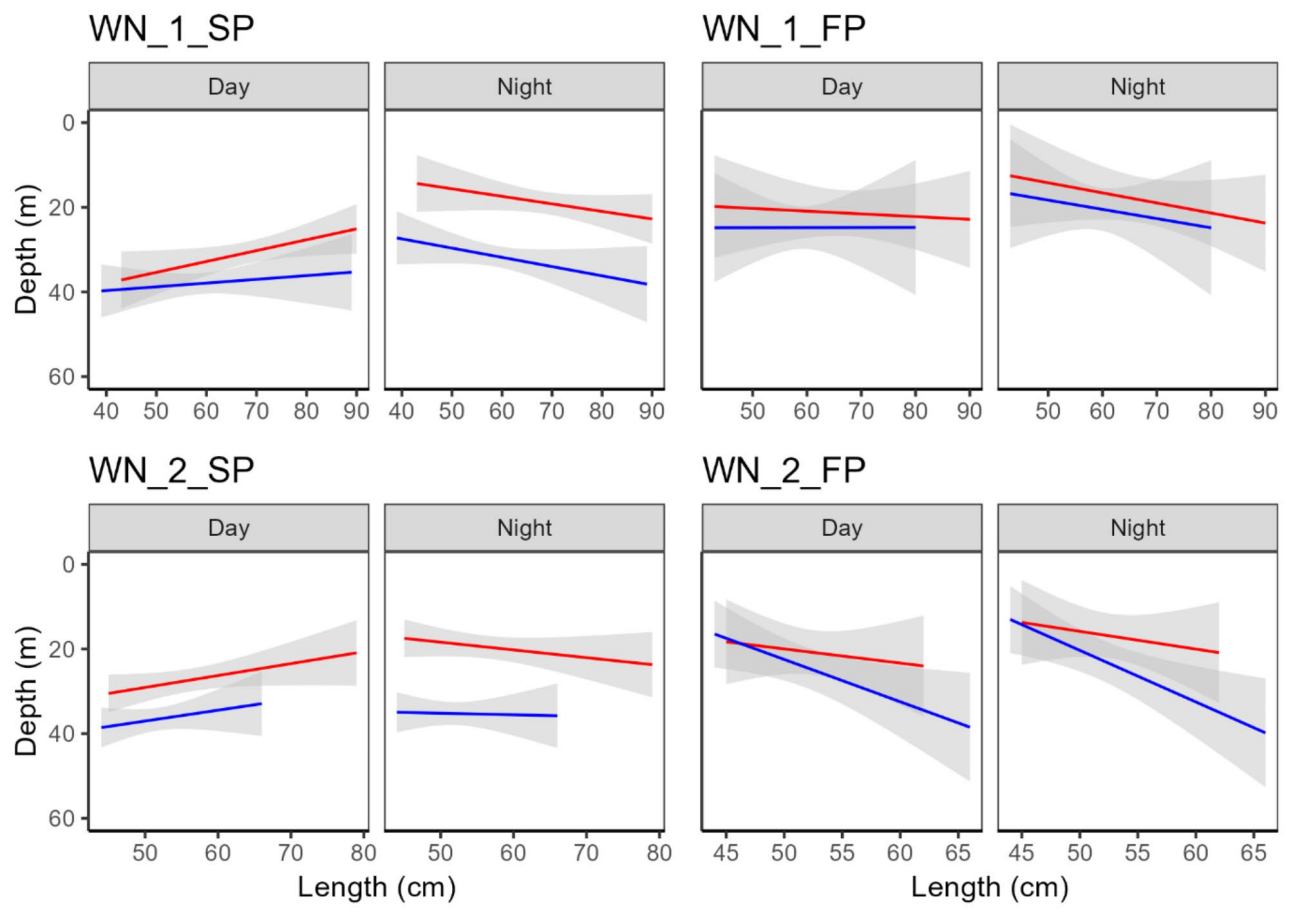
Overall depth differences at the western Norway spawning grounds as a function of sex (red: female; blue: male), fish length (cm), day/night and spawning period (SP, spawning and FP: feeding). The shaded area indicates model uncertainty (± 95% confidence intervals) for the continuous fixed effect length.

**Fig. 3:**
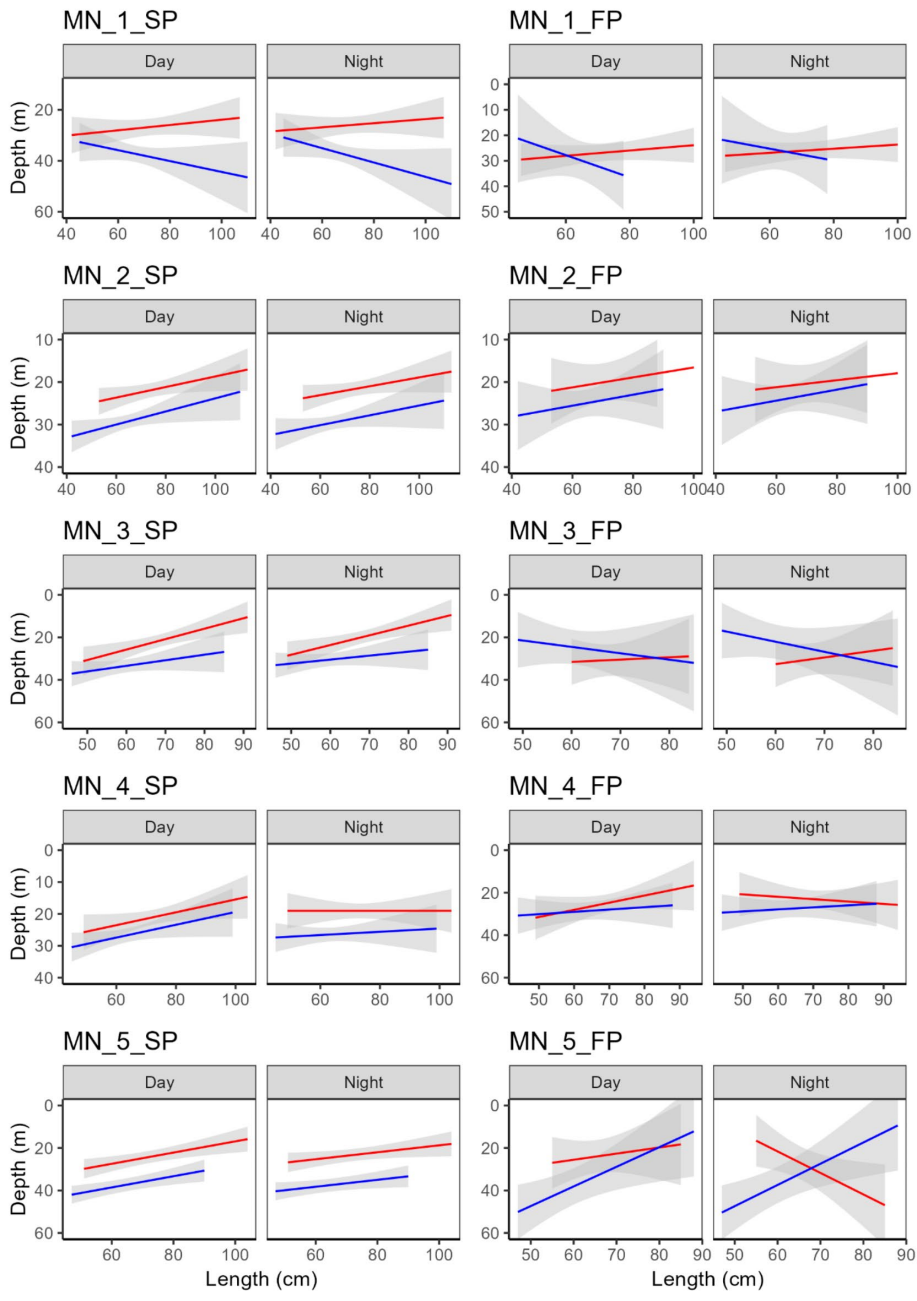
Overall depth differences at the mid Norway spawning grounds as a function of sex (red: female; blue: male), fish length (cm), day/night and spawning period (SP, spawning and FP: feeding). Shaded area indicates model uncertainty uncertainty (± 95% confidence intervals) for the continuous fixed effect length.

During the spawning period, males stayed overall significantly deeper than females at all 7 of the spawning grounds across the range of fish sizes examined (Figs. 2 and 3; Tables 2, 3 and 4). The most pronounced difference in depth between sexes was at MN_5 (β = 11.12, *p* < 0.001, Fig. 3). In contrast, this pattern was much less consistent during the foraging period, when it tended to depend on fish size (Figs. 2 and 3; Tables 2, 3 and 4). Overall, males were not significantly deeper than females during feeding periods at 6 of the 7 sites examined (*p* values from to 0.307 to 0.839, Tables 2, 3 and 4).

**Table 2:** Model summaries for the western Norway spawning grounds overall depth distribution during the spawning (sp) and feeding (fp) periods. The response variable was depth for all models. The default treatment contrast in R was used, with the intercept value epresenting females during the day. Square brackets indicate categorical variables, i.e. males and night, tested against this reference value.Fixed effects; length = length centered – continous variable, sex = sex of fish – categorical variable, DN = day or night – categorical variable. X indicates interaction. The values for the fixed effects are shown at the top of the table with the random effects underneath.

*Predictors*	WN_1_sp			WN_1_fp			WN_2_sp			WN_2_fp		
	*Estimates*	*CI*	*p*	*Estimates*	*CI*	*p*	*Estimates*	*CI*	*p*	*Estimates*	*CI*	*p*
(Intercept)	32.21	29.21–35.21	**< 0.001**	21.11	15.41–26.81	**< 0.001**	28.39	25.46–31.31	**< 0.001**	20.18	14.49–25.87	**< 0.001**
Length	-0.26	-0.50 – -0.01	**0.038**	0.06	-0.38–0.51	0.776	-0.28	-0.60–0.04	0.082	0.33	-0.80–1.46	0.561
Sex [M]	5.49	1.65–9.34	**0.005**	3.69	-4.35–11.72	0.368	8.00	4.16–11.84	**< 0.001**	2.88	-4.46–10.21	0.442
DN [Night]	-14.38	-14.47 – -14.30	**< 0.001**	-3.79	-3.86 – -3.72	**< 0.001**	-9.53	-9.66 – -9.40	**< 0.001**	-4.11	-4.18 – -4.04	**< 0.001**
Length × Sex [M]	0.17	-0.21–0.55	0.381	-0.07	-0.92–0.79	0.879	0.03	-0.57–0.62	0.933	0.67	-0.74–2.07	0.352
Length × DN[Night]	0.43	0.43–0.44	**< 0.001**	0.17	0.17–0.18	**< 0.001**	0.46	0.45–0.48	**< 0.001**	0.08	0.07–0.10	**< 0.001**
Sex [M] × DN[Night]	9.02	8.90–9.13	**< 0.001**	0.15	0.04–0.26	**0.006**	8.42	8.26–8.59	**< 0.001**	2.09	1.96–2.21	**< 0.001**
Length × Sex [M] × DN[Night]	-0.13	-0.14 – -0.12	**< 0.001**	0.05	0.03–0.06	**< 0.001**	-0.17	-0.20 – -0.15	**< 0.001**	0.13	0.10–0.16	**< 0.001**
**Random Effects**												
σ^2^	107.78			112.26			66.71			51.96		
τ_00_	13.67 _Year: Jday_			31.53 _Year: Jday_			21.77 _Year: Jday_			40.74 _Year: Jday_		
	88.85 _Serial2_			20.18 _Jday_			1.37 _Jday_			50.08 _Jday_		
	5.19 _Jday_			205.49 _Serial2_			35.93 _Serial2_			89.60 _Serial2_		
ICC	0.50			0.70			0.47			0.78		
N	119 _Serial2_			60 _Serial2_			42 _Serial2_			29 _Serial2_		
	3 _Year_			3 _Year_			3 _Year_			3 _Year_		
	60 _Jday_			246 _Jday_			60 _Jday_			246 _Jday_		
Observations	605,291			887,411			190,250			358,737		
Marginal R^2^ / Conditional R^2^	0.197 / 0.599			0.020 / 0.702			0.286 / 0.621			0.040 / 0.785		

**Table 3:** Model summaries for the mid Norway spawning grounds during the spawning period. The response variable was depth for all models. The treatment contrast of R was used with the intercept value representing females during the day. Square brackets indicate categorical variables, i.e. males and night, tested against this reference value. Fixed effects; length = length centered - continous variable, sex = sex of fish - categorical variable, DN = day or night - categorical variable. X indicates interaction. The values for the fixed effects are shown at the top of the table with the random effects underneath.

*Predictors*	MN_1			MN_2			MN_3			MN_4			MN_5		
	*Estimate*	*CI*	*p*	*Estimate*	*CI*	*p*	*Estimate*	*CI*	*p*	*Estimate*	*CI*	*p*	*Estimate*	*CI*	*p*
(Intercept)	27.59	23.96–31.23	**< 0.001**	22.83	20.88–24.78	**< 0.001**	24.61	21.08–28.14	**< 0.001**	22.99	19.81–26.17	**< 0.001**	25.84	22.78–28.89	**< 0.001**
Length	-0.10	-0.32–0.11	0.338	-0.12	-0.25 – -0.00	**0.046**	-0.49	-0.80 – -0.19	**0.002**	-0.20	-0.41–0.01	0.058	-0.26	-0.43 – -0.10	**0.002**
Sex [M]	9.19	4.48–13.91	**< 0.001**	6.15	3.70–8.59	**< 0.001**	8.18	3.59–12.77	**< 0.001**	3.90	0.09–7.70	**0.045**	11.12	6.95–15.29	**< 0.001**
DN [Night]	-1.06	-1.16 – -0.97	**< 0.001**	-0.44	-0.49 – -0.40	**< 0.001**	-2.06	-2.17 – -1.95	**< 0.001**	-3.95	-4.08 – -3.83	**< 0.001**	-1.54	-1.62 – -1.46	**< 0.001**
Length× Sex[M]	0.32	-0.06–0.70	0.102	-0.03	-0.22–0.16	0.758	0.23	-0.24–0.70	0.342	0.00	-0.29–0.29	0.996			
Length×DN [Night]	0.02	0.02–0.03	**< 0.001**	0.02	0.02–0.02	**< 0.001**	0.04	0.02–0.05	**< 0.001**	0.20	0.19–0.21	**< 0.001**	0.10	0.10–0.10	**< 0.001**
Sex [M] × DN [Night]	0.56	0.45–0.68	**< 0.001**	0.84	0.79–0.89	**< 0.001**	-0.71	-0.86 – -0.56	**< 0.001**	3.56	3.42–3.70	**< 0.001**	1.84	1.73–1.95	**< 0.001**
LC × Sex[M] × DN [Night]	0.05	0.04–0.05	**< 0.001**	0.02	0.01–0.02	**< 0.001**	0.04	0.02–0.06	**< 0.001**	-0.05	-0.06 – -0.04	**< 0.001**			
**Random Effects**															
σ^2^	59.32			24.62			82.44			50.25			50.77		
τ_00_	16.61 _Year: Jday_			1.68 _Year: Jday_			14.87 _Year: Jday_			5.25 _Year: Jday_			6.80 _Year: Jday_		
	189.52 _Serial_			54.74 _Serial_			94.51 _Serial_			97.26 _Serial_			104.76 _Serial_		
	11.12 _Jday_			3.29 _Jday_			7.99 _Jday_			1.78 _Jday_			14.19 _Jday_		
ICC	0.79			0.71			0.59			0.67			0.71		
N	154 _Serial_			173 _Serial_			90 _Serial_			159 _Serial_			105 _Serial_		
	3 _Year_			3 _Year_			3 _Year_			3 _Year_			3 _Year_		
	77 _Jday_			78 _Jday_			78 _Jday_			77 _Jday_			77 _Jday_		
Observations	317,165			734,706			292,472			284,122			301,756		
Marginal R^2^ / Conditional R^2^	0.086 / 0.804			0.164 / 0.756			0.162 / 0.654			0.072 / 0.698			0.198 / 0.769		

**Table 4:** Model summaries for the mid Norway spawning grounds during the feeding period. The response variable was depth for all models. The treatment contrast of R was used with the intercept value representing females during the day. Square brackets indicate categorical variables, i.e. males and night, tested against this reference value. Fixed effects; length = length centered – continous variable, sex = sex of fish – categorical variable, DN = day or night – categorical variable. X indicates interaction. The values for the fixed effects are shown at the top of the table with the random effects underneath.

*Predictors*	MN_1			MN_2			MN_3			MN_4			MN_5		
	*Estimate*	*CI*	*p*	*Estimate*	*CI*	*p*	*Estimates*	*CI*	*p*	*Estimates*	*CI*	*p*	*Estimates*	*CI*	*p*
(Intercept)	29.11	21.00–37.22	**< 0.001**	21.16	15.50–26.83	**< 0.001**	31.47	21.19–41.75	**< 0.001**	27.62	21.99–33.26	**< 0.001**	25.90	16.13–35.66	**< 0.001**
Length	-0.28	-0.85–0.30	0.351	-0.12	-0.48–0.25	0.532	-0.11	-1.10–0.88	0.830	-0.34	-0.79–0.12	0.147	-0.29	-1.19–0.61	0.528
Sex [Male]	-1.19	-12.64–10.26	0.839	4.30	-2.29–10.90	0.201	-6.77	-19.75–6.22	0.307	1.25	-5.69–8.20	0.724	13.31	0.30–26.31	**0.045**
DN [Night]	-1.08	-1.16 – -1.00	**< 0.001**	-0.02	-0.06–0.02	0.402	0.89	0.75–1.04	**< 0.001**	-5.53	-5.65 – -5.41	**< 0.001**	-5.47	-5.56 – -5.38	**< 0.001**
Length × Sex[M]	0.71	-0.28–1.71	0.161	-0.01	-0.51–0.49	0.963	0.41	-0.92–1.74	0.545	0.23	-0.37–0.83	0.460	-0.64	-1.78–0.50	0.275
Length × DN [Night]	0.04	0.03–0.05	**< 0.001**	0.03	0.03–0.04	**< 0.001**	-0.20	-0.23 – -0.18	**< 0.001**	0.45	0.44–0.46	**< 0.001**	1.30	1.28–1.32	**< 0.001**
Sex [M] × DN [Night]	-1.50	-1.61 – -1.39	**< 0.001**	-1.18	-1.22 – -1.13	**< 0.001**	-3.20	-3.36 – -3.03	**< 0.001**	4.39	4.27–4.52	**< 0.001**	4.85	4.58–5.12	**< 0.001**
Length× Sex[M] × DN [Night]	-0.24	-0.26 – -0.23	**< 0.001**	-0.04	-0.04 – -0.03	**< 0.001**	0.38	0.35–0.40	**< 0.001**	-0.43	-0.45 – -0.42	**< 0.001**	-1.38	-1.41 – -1.34	**< 0.001**
**Random Effects**															
σ^2^	50.95			20.60			66.23			26.42			51.49		
τ_00_	19.16 _Year: Jday_			1.95 _Year: Jday_			69.18 _Year: Jday_			18.77 _Year: Jday_			15.08 _Year: Jday_		
	0.00 _Jday_			1.34 _Jday_			4.51 _Jday_			9.86 _Jday_			3.59 _Jday_		
	334.36 _Serial_			114.29 _Serial_			198.60 _Serial_			130.21 _Serial_			180.13 _Serial_		
ICC				0.85			0.80			0.86			0.79		
N	47 _Serial_			65 _Serial_			26 _Serial_			61 _Serial_			22 _Serial_		
	3 _Year_			3 _Year_			3 _Year_			3 _Year_			3 _Year_		
	214 _Jday_			214 _Jday_			214 _Jday_			214 _Jday_			214 _Jday_		
Observations	301,031			885,197			280,933			301,169			144,652		
Marginal R^2^ / Conditional R^2^	0.185 / NA			0.045 / 0.858			0.057 / 0.816			0.023 / 0.861			0.216 / 0.839		

The vertical separation between sexes during spawning periods was also significantly influenced by diel period (day/night) at all five spawning grounds, but this trend was more obvious in Western Norway (Sex x DN β coefficients from 8.42 to 9.02, *p* values < 0.001) than Mid Norway (Sex x DN β coefficients from − 0.71 to 3.56, p values < 0.001). At all 7 spawning grounds there was a statistically significant, albeit variable, influence of fish size on diel vertical separation between sexes (Length x DN x Sex β coefficients from − 1.38 to 0.38, p values < 0.001) (Figs. 2 and 3; Tables 2, 3 and 4). At all 7 spawning grounds, larger females were shallower than smaller females during the day (Figs. 2 and 3), whereas the influence of size on depth was more variable at night. Similarly, at 6 of the 7 spawning grounds larger males were shallower than smaller males during the day (although the opposite pattern was true at MN1_SP, Fig. 3). and the influence of fish size on depth was more variable at night.

## Periodic descents – spawning period

A subset of 321 data-rich individuals (i.e. individuals with > 2000 detections and > 20 detection days) from February and March in western Norway and February to April in mid Norway were selected for in-depth analyses of spawning behaviour (N_western Norway_ = 96, N_mid Norway_ = 225, Table 1). For western Norway the average number of detections days was 49.1 (± 1.25 standard error (SE)), and the average number of detections was 7675 (± 375 SE). The average number of bursts per fish, with a new burst starting if the fish had gone undetected for > 24 h, was 2.2 (± 0.2 SE). Average male and female depth were 35.3 m (± 0.97 SE) and 21.9 m (± 0.62 SE), respectively. For mid Norway the average number of detections days was 55.2 (± 1.2 SE), and the average number of detections was 7645 (± 290 SE). The average number of bursts per fish was 3.4 (± 0.20 SE). Average male and female depth were 34.2 m (± 0.82 SE) and 23.8 m (± 0.93 SE), respectively.

**Fig. 4:**
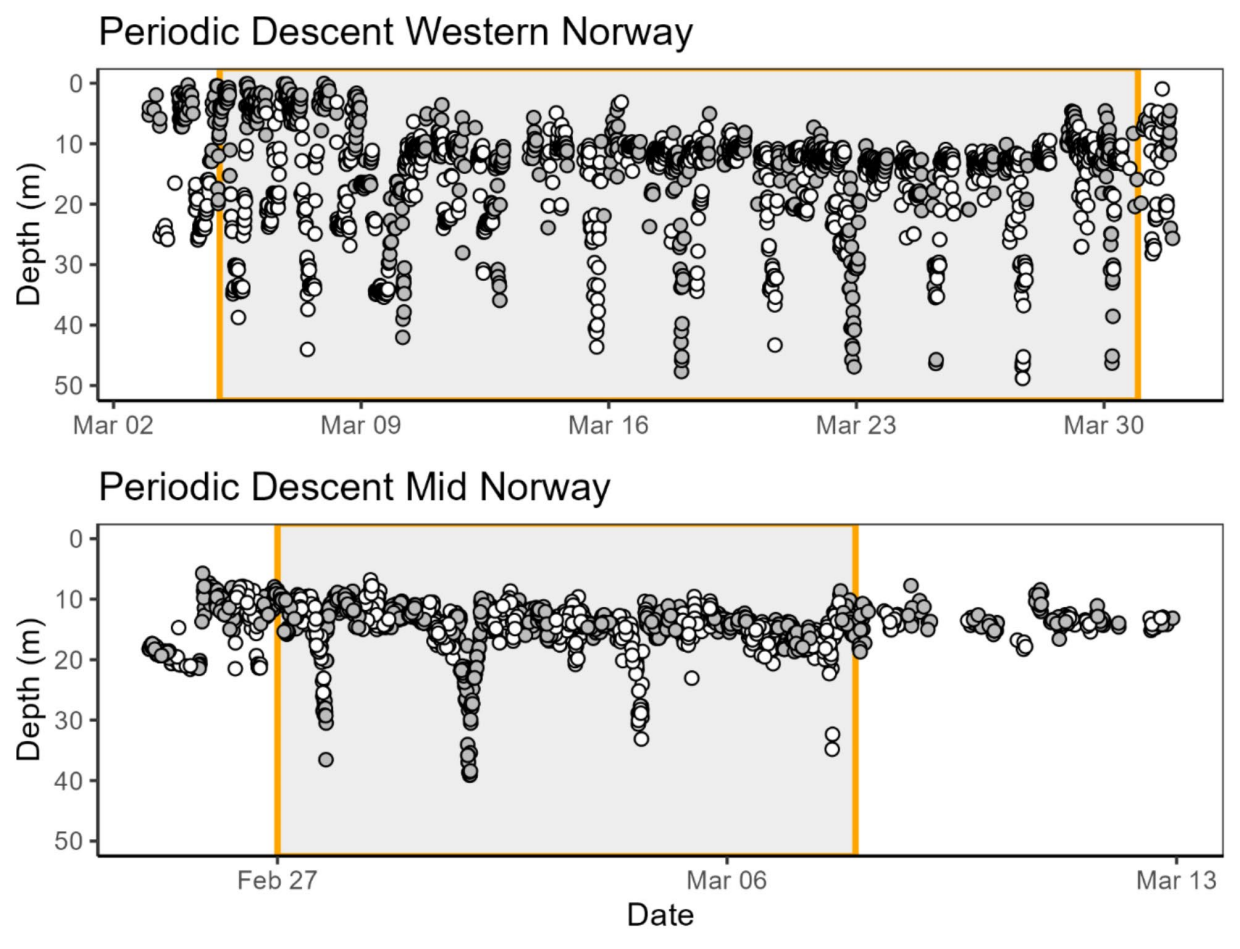
Example of Periodic Descents showing one female from the western (WN) and mid Norway (MN) spawning grounds. Shaded area indicates the onset and end of the behavioural sequence as identified by our code. White fill = day, grey fill = night.

**Fig. 5:**
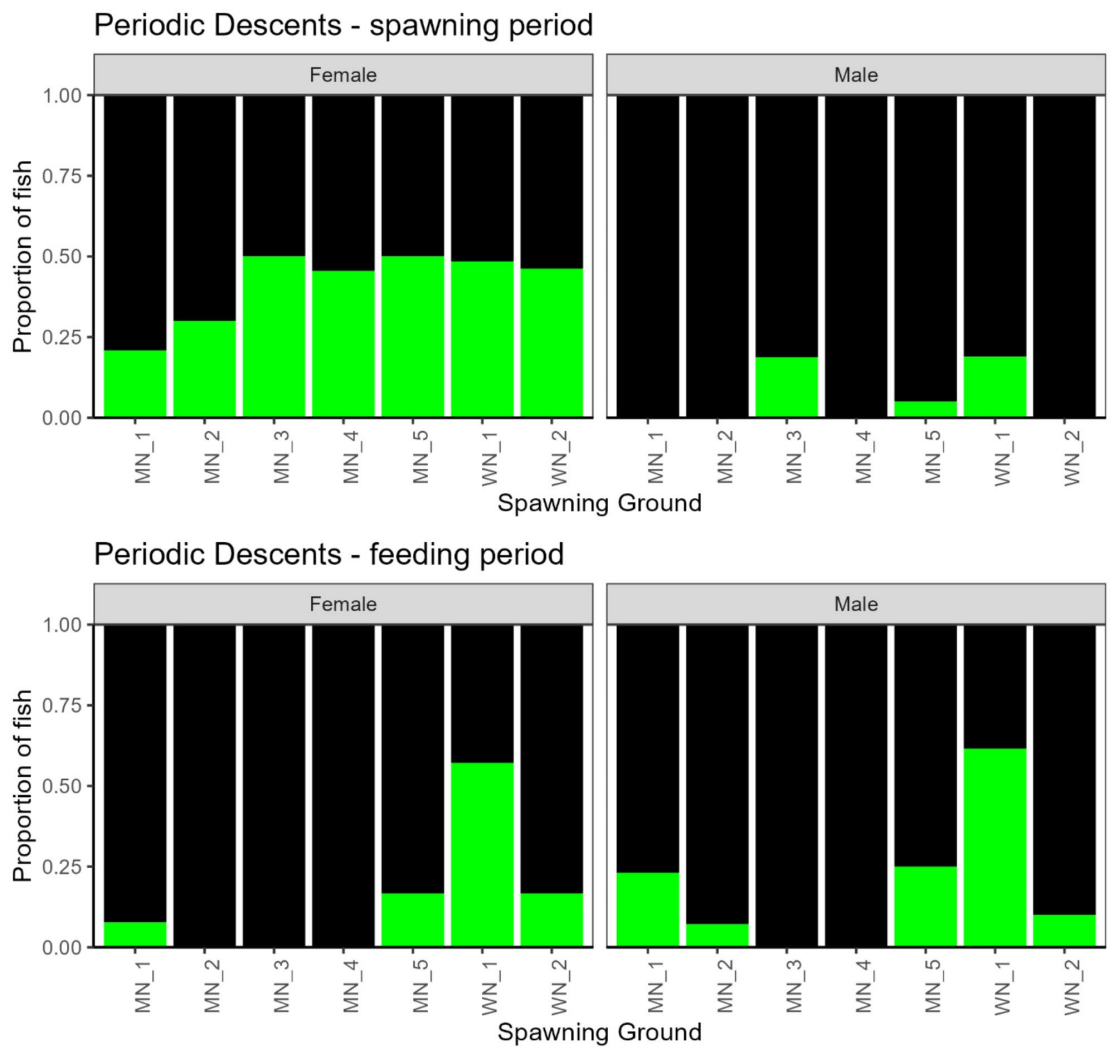
Proportion of male and female cod fish performing periodic descents at each study ground across years for the spawning period (upper panels) and the feeding period (lower panels), with the latter represented with the months June - August. Green fill respresents the porportion of cod displaying periodic decsents.

Between 25 and 55% of the females at each spawning ground performed periodic descents (*n* = 51), compared to 0–20% of males (*n* = 11) (Figs. 4 and 5). Accordingly, sex was a strongly significant predictor of periodic descents in the final model (Table 5, *p* < 0.00001). Body length, although not significant, was estimated to have a slight non - significant positive effect and was retained in the final model (Table 5). The total variation explained by the fixed effects in the model was 29% (Table 5).

**Table 5:** Model results showing the explanatory variables and their corresponding significance levels for all variables retained in the final models for periodic descents during the spawning and feeding period, e.g. the months August to September for the latter period. The response variable in both tests was the binomial variable periodic descents with a value of 1 representing fish that had per**f**ormed this behaviour and 0 respesetning fish that had not performed the behaviour. The treatment contrast of R was used with the intercept value representing the value for females. Square brackets indicate the categorical variable sex, i.e. males, tested against this reference value. The top part of the model shows the results for the fixed effects, the bottom part shows the random effects.

*Predictors*	Periodic descents - sp			Periodic descents - fp		
	*Log-Odds*	*CI*	*p*	*Log-Odds*	*CI*	*p*
(Intercept)	-1.47	-3.30–0.36	0.115	-2.39	-3.83 – -0.95	**0.001**
Length/sd(Length)	0.19	-0.13–0.51	0.240			
Sex [Male]	-2.33	-3.06 – -1.60	**< 0.001**	0.39	-0.62–1.40	0.450
**Random Effects**						
σ^2^	3.29			3.29		
τ_00_	0.32 _Sp_Ground_			1.88 _Sp_Ground_		
ICC	0.09			0.36		
N	7 _Sp_Ground_			7 _Sp_Ground_		
Observations	320			135		
Marginal R^2^ / Conditional R^2^	0.290 / 0.352			0.007 / 0.368		

**Fig. 6:**
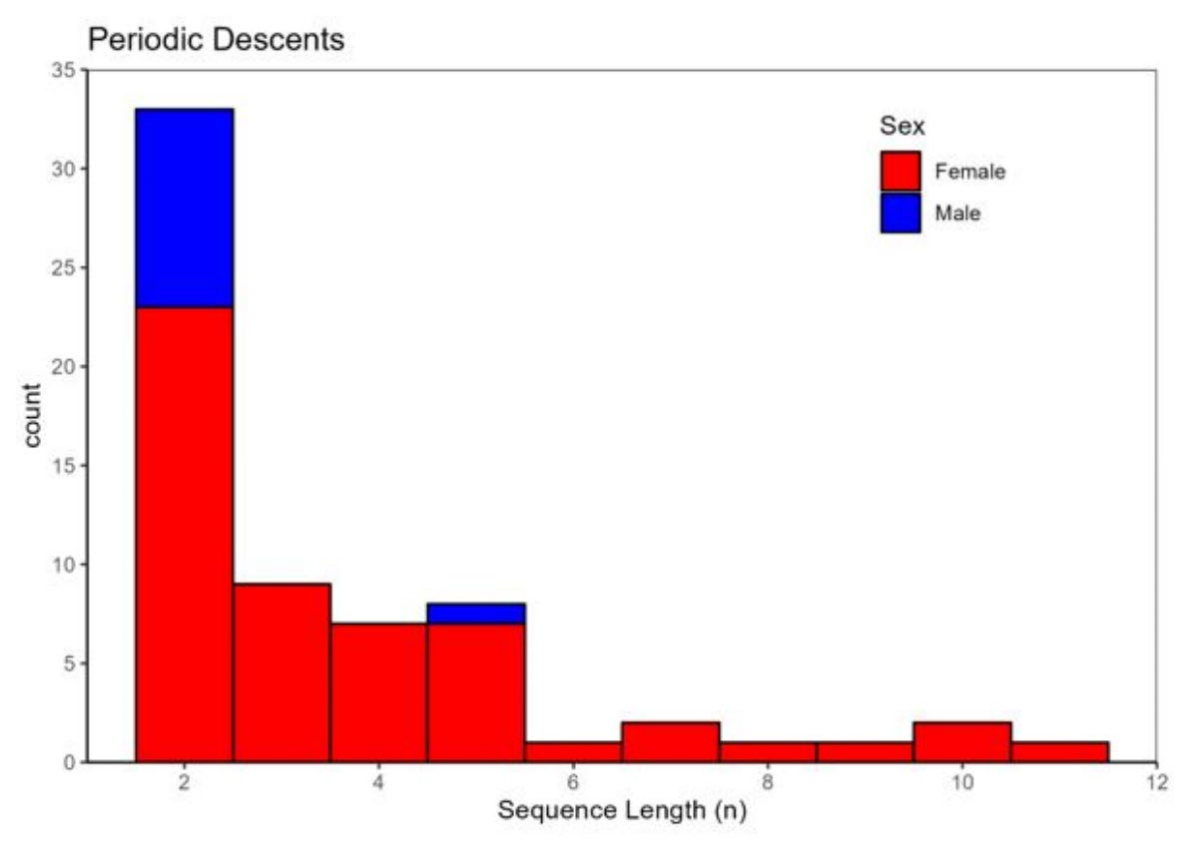
Distribution of sequence lengths, i.e. the number of decsents whithin a sequence, for periodic descents for males and females.

**Fig. 7:**
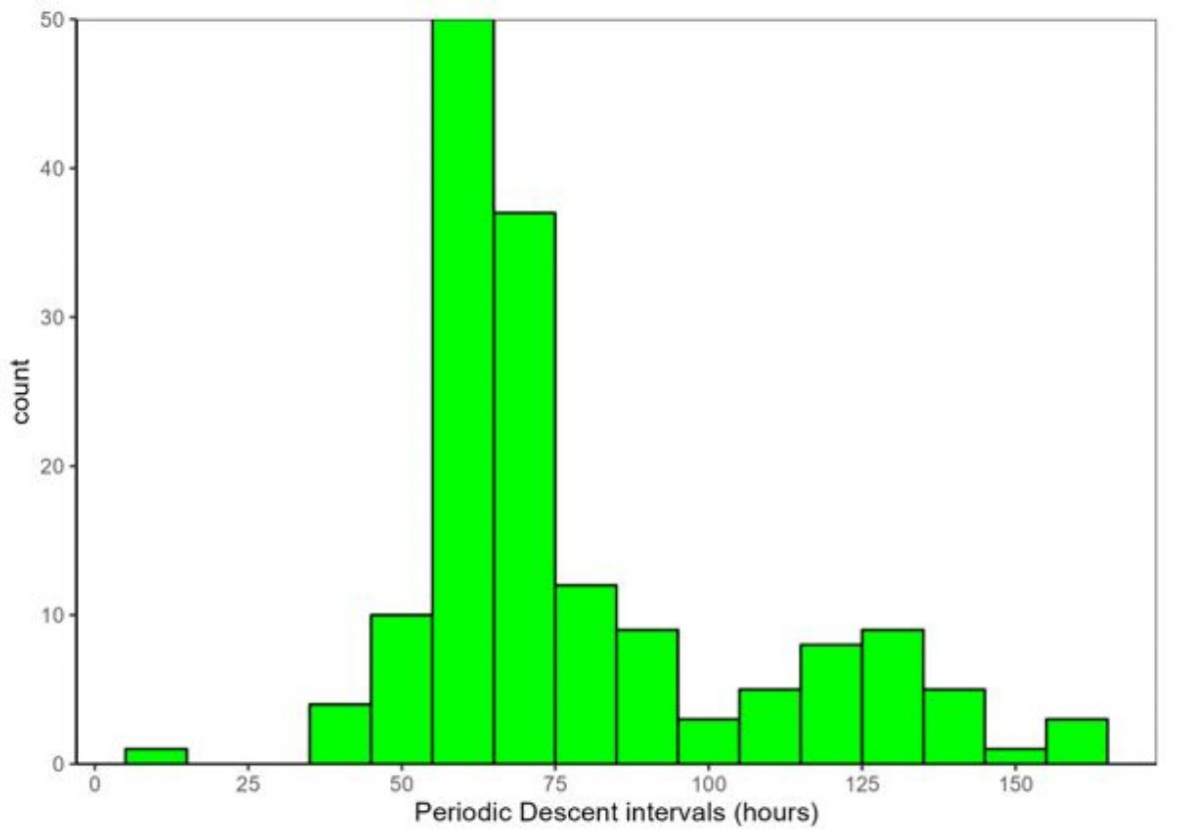
Frequency distribution of intervals between periodic descents within valid sequences for females during the spawning period.

**Fig. 8:**
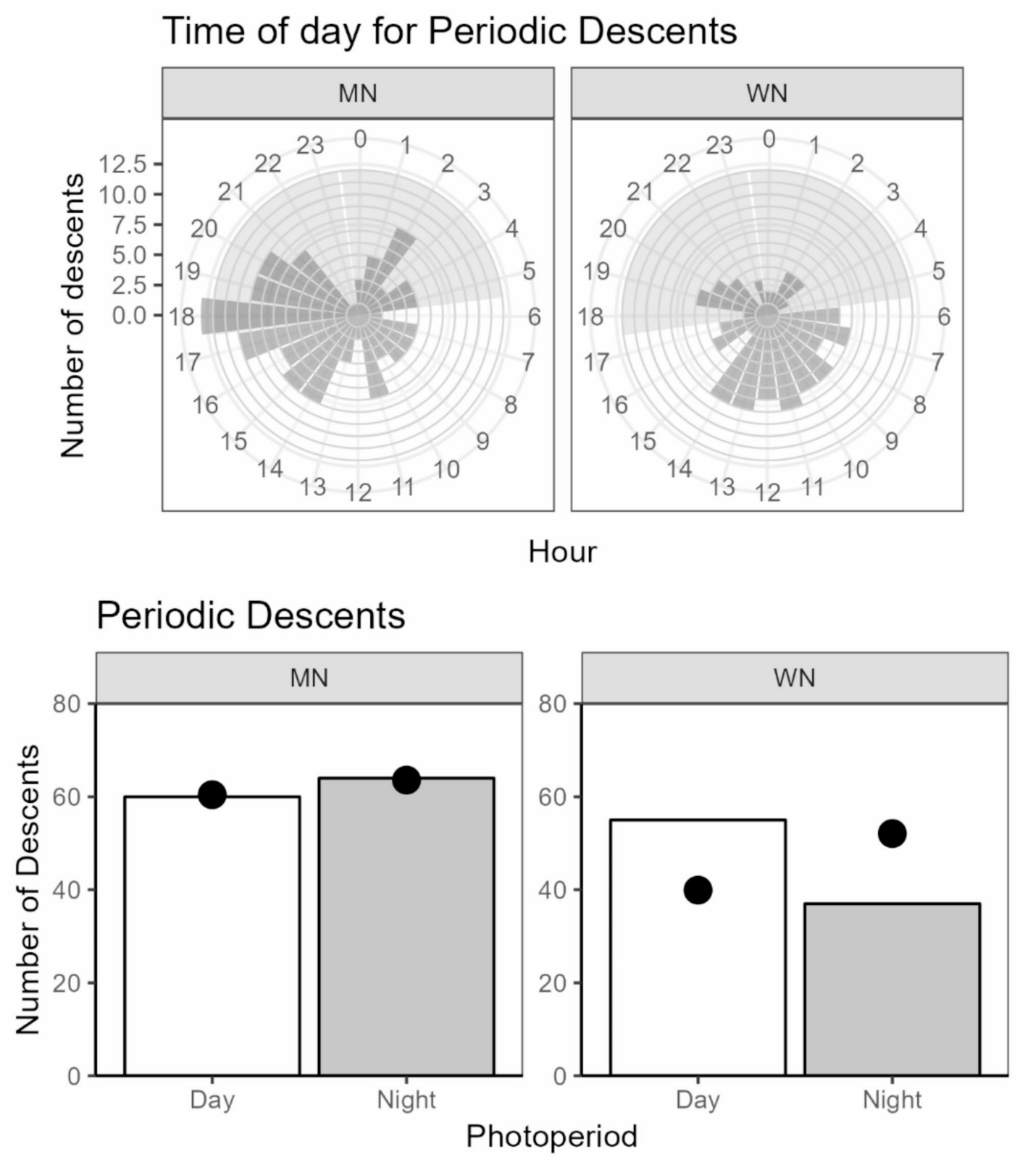
The time of day when periodic descents occurred in UTC time, top panels, and related to the photoperiod, bottom panels for females during the spawning periods. Shading indicates approximate nighttime hours on the median day of the spawning period. In the bottom panels circles indicate expected number of descents during night and day if their timing was randomly distributed, based on the photoperiod lengths of day and night for March 1 (Western Norway) and March 15 (Mid Norway), i.e. the median calendar day of the spawning period in each area.

The recorded sequence lengths varied between 2 and 11 periodic descents. Notably, all but one of the sequences involving more than two descents were undertaken by females (Fig. 6). Considering the females only, there was a striking modality in the interval between these descents (Fig. 7). By far the most common descent interval was between 60 and 70 h (~ 60%, *n* = 157), with a median interval of 67.8 h. A second smaller peak was observed between 120 and 140 h (Fig. 7). In both study areas descents were observed both during day and nighttime (Fig. 8), but the latter was significantly more common in mid Norway (lmer - model, β = − 0.63, z = -2.14, *p* < 0.05). The average depth of “periodic descent” females was 21.3 m (± 0.76 SE) and 18.3 m (± 0.62 SE) in western and mid Norway respectively, whereas females not undertaking this behaviour had an average depth of 22.4 m (± 0.49 SE) and 26.8 m (± 0.93 SE) in western and mid Norway, respectively. The average maximum depth recorded when a female undertook a periodic descent was 48.3 m (± 2.4 SE) in western Norway and 41.6 m (± 2.1) in mid Norway.

All the eight different scenarios tested during the spawning period by modifying the selection criteria for denoting fish as a periodic descent fish showed a clear female bias (Supplementary Table 1). Relaxing the times allowed between descents only marginally increased the number of fish classified as performing periodic descents, whereas increasing the maximum depth criteria, especially, and the minimum number of descents required within a sequence reduced the proportion of fish performing the periodic descent behaviour substantially (Supplementary Table 1).

### Periodic descents – feeding period

During the control feeding period, i.e. June to August, a subset of 135 data-rich individuals were selected for in - depth behavioural analyses (N_western Norway_ = 48, N_mid Norway_ = 87, Table 1). In western Norway the average number of detections days was 77.0 (± 2.7 SE), and the average number of detections was 10 528 (± 892 SE), whereas the average number of bursts per fish was 3.1 (± 0.36 SE). Average male and female depth were 23.4 m (± 1.3 SE) and 25.5 m (± 1.1 SE), respectively. In mid Norway the average number of detections days was 74.3 (± 2.5 SE), and the average number of detections was 10 962 (± 736 SE). The average number of bursts per fish was 2.7 (± 0.37 SE). Average male and female depth were 29.3 m (± 1.4 SE) and 27.0 m (± 1.7 SE), respectively.

The proportion of females performing periodic descents was overall much reduced or absent during this time compared to the spawning period except for WN_1 (Fig. 6). Sex was retained as an explanatory variable in the final model, but was not significant (Table 5, *p* = 0.45) and less than 1% of the variation was explained by the final selected model (Table 5).

## Discussion

Males occupied deeper positions than females during spawning seasons across seven different spawning grounds from western to mid Norway. Together with earlier studies, the extensive dataset analysed in the present study reinforces the importance of the vertical dimension to the mating system of cod in coastal Norwegian habitats. We also detected clear sex-specific behaviours that lend further support to the hypothesis that the cod mating system conforms to a lek-like structure. Specifically, an untested prediction from previous studies is that females only enter the male-dominated aggregation when they are ready to spawn^16,25^. Our study provides the strongest evidence to date in support of this hypothesis, with most females descending to the depth of the males at intervals of 3–4 days, closely aligning with inter-batch intervals reported for cod spawning in captivity^9,10^.

### Overall depth

Sex-specific depth use by cod during the spawning season has also been noted in earlier telemetry studies^19,20^and it has been suggested that females typically occupy peripheral positions and move between male aggregations^18,46^. Interestingly, Brawn^5^also writes in her classical paper that Sars (1865) records that “the male fish during the act of spawning generally swims deeper than the female”. In contrast, a tendency for females to stay deeper were found at cod spawning grounds in the western Atlantic^17,26^. Similarly, in Icelandic waters, different populations, but apparently not sexes within populations, separate by depth at spawning grounds^47^.

An earlier study on Atlantic cod at one of the western Norway spawning grounds also found that the vertical separation between sexes was larger during night than during the day^19^. This pattern is consistent with our findings in western Norway, although not for the mid Norway spawning grounds. In addition, the influence of fish size on depth use was not consistent. Larger fish were sometimes found at deeper positions and sometimes shallower positions compared to smaller fish depending on spawning grounds, sex and between the night and day. These variable results likely reflect that the spawning behaviour is not fixed but instead comprised of plastic traits, where the optimal trade-offs will be shaped by both the abiotic and social environment^48,49^.

### Periodic descents

We acknowledge that our criteria for detecting female spawning events, i.e. the periodic decent behaviour, is unlikely to be perfect as it is based on interpretation of movement data rather than direct observations of spawning activity. Also, although generally much less prevalent for females, some fish outside the putative spawning period were also classified as performing this behaviour. The observation of periodic descents thus needs to be combined with knowledge of the spawning period and preferably information on the general depth distribution patterns of the sexes before inferring spawning activity.

Taken in the context of previous knowledge on cod spawning behaviour from laboratory studies, our study presents novel insights on cod spawning in natural environments and represents a useful reference point for future studies. Our finding that female cod descend towards deeper waters at intervals of several days is consistent with the lek-description of the cod mating system and females having a documented batch spawning strategy^9,10^. From this perspective, it is likely that the female descents observed here represent spawning activity, where the females move towards male aggregations located closer to the seafloor. We note that some males also matched our selection criteria, but there was nevertheless a strong bias towards females. In particular, individuals displaying more than two periodic descents were almost exclusively female.

Some individuals were tracked across more than one spawning season. We acknowledge that our initial data filtering, where only the spawning season immediately following tagging was included in the analyses, involves both positives and drawbacks. One obvious drawback is that we miss information about the level of repeatability of spawning behaviour, an interesting topic for further studies. However, a key advantage of our approach was that an accurate measurement of body length could be included as a relevant covariate in the analyses.

We found a striking periodicity in the intervals between female periodic descents indicative of spawning events, with a distinct peak between 60 and 70 h using the maximum depth during a periodic descent as a proxy for the time of female spawning. Cod batch intervals are temperature dependent, with shorter intervals at higher temperatures and a reported value of 70 h between batches at 5.5 ℃^9,10^. A string of temperature loggers from the surface to the bottom was placed at a central location at each spawning ground at both our study locations, continuously logging temperature every 2 h. In both mid and western Norway the water column was generally well-mixed with little variation during the spawning period with a typical temperature range of 5.5–6.5 and 6.5–8 ℃, respectively (unpublished data). The observed intervals thus closely match the expected batch intervals at this ambient temperature. The smaller peak around 120–140 h may be due to a batch not being recorded. The female descents depth was somewhat deeper than average male depths, but within the normal depth range of males, i.e. around the 60–80% quantile of male depths in both areas. In sum, both the female bias in the individuals performing periodic descents, the interval lengths, and the depths of the periodic descents agree well with an expected pattern of female cod spawning events.

If all spawning events for a female occur during a periodic descent event, and all such events are captured within a sequence, sequence lengths (Fig. 7) will equate to the number of batches released by individual females. The longest sequence lengths in our dataset were 9–11 periodic descents, which could be close to the maximum for wild cod in these study areas, even though this is considerably less than that reported for well-fed highly fecund cod in experimental studies that may release upwards of 20 batches^9,10^. The secondary peak in the inter-spawning interval reported presently (Fig. 7) suggests that some spawning events may have been missed, as would be expected if for example the fish spawned outside of the detection range of the receivers or if some spawning events did not leave a clear signal in the acoustic telemetry data. This could, for instance depend on the topography of the spawning seascape, where some sites may be difficult to monitor, and other sites may not require much vertical swimming activity. Similarly, for females with the shortest sequence lengths, i.e. 2–3 descents, some batches may have been missed.

In our study, female descents were not restricted to any specific photoperiod and occurred both during the day and night. Previous studies on cod have reported predominant night-time spawning inferred from either egg staging^50^or spatial movement of cod^26^. Night-time spawning and perhaps particularly crepuscular, or dusk, spawning is common in aquatic animals (e.g^51,52^). Within the mid Norway study area, newly spawned cod eggs have been found during the middle of the day, unequivocally showing that daytime spawning does occur here^53^. Similarly, cod drumming sounds, associated with mating behaviour and spawning^6,54^, were more commonly heard during the day in a recent field study in the Gulf of Maine and New England^55^and indeed also heard during the day (night was not investigated in this study) in 2019 at the WN_1 spawning ground^56^. The indication of more daytime spawning in western Norway compared to mid Norway is intriguing and an interesting topic for further investigations. Given the increasing evidence that peak cod spawning may not consistently occur during dusk/night, as previously assumed, further investigation into the diel distribution of cod spawning activity across its geographical range is encouraged.

Finally, the percentage of data rich females performing periodic descents varied from 25 to 52% across spawning grounds. Some females may not have been spawning when they were detected at the monitored spawning grounds, instead shedding their eggs at nearby spawning grounds shortly before or after this period. Even so, this also indicates that not all females perform such pronounced descents when spawning. Consistent with this interpretation, non - descending females occupied deeper positions in the water column than the periodic descent females, especially so in mid Norway, and hence may approach and retreat from the lek horizontally.

### Concluding remarks

In conclusion, the present study highlights the importance of vertical movement behaviours during cod spawning along the Norwegian coastline. It is interesting - and inspiring for future studies - to note that the sex- specific depth patterns presented here, i.e. males staying deeper than females, appears not to be found in the western Atlantic^26^and possibly also Icelandic waters^47^, highlighting the dynamic nature of fish spawning behaviour, and consequently the need for a population-based approach in the study of cod mating strategies. The presently observed sex-specific periodic descents correspond well to the lek-like description of the cod mating system and with cod reproductive physiology, where females are known to mature and release batches of eggs at intervals of several days as part of a bet-hedging strategy.

## Electronic supplementary material

Below is the link to the electronic supplementary material.

Supplementary Material 1

## Data Availability

The data for this study is stored at the Institute of Marine Research and can be made available by the authors to any qualified researcher upon reasonable request.
