# Supplementary material for: Sex-specific vertical movements of spawning atlantic cod in coastal habitats inferred from acoustic telemetry
Source: Sci Rep. 2024 Oct 6;14:23242. doi: 10.1038/s41598-024-74896-2 (PMC11455899; doi:10.1038/s41598-024-74896-2)
Supplement: Supplementary file 1 — Supplementary Material 1 [file 41598_2024_74896_MOESM1_ESM.docx]

**Sex-specific vertical movements of spawning Atlantic cod in coastal habitats inferred from acoustic telemetry**

Skjæraasen JE, Olsen EM, McQueen K, Nyqvist D, Meager JJ, Karlsen Ø, Sivle LD

**Supplementary Table 1.** Summary results for the scenario testing using different selection criteria (PD_Method) for depth, time allowed between batces and the minimum number of batches required for cod to be denoted as a “periodic descent” cod. Under PD_Method the first number refers to how much deeper the maximum depth (in percent) than the average of the maximum depth day before and after must be, i.e. 50 or 100 %, the second number represent time allowed between batches, i.e. 6 or 10 days, and the last number the number of batches required to form a valid sequence, i.e. 2 or 3. 100_6_2 thus denotes a selection criteria of 100 % deeper, 6 days maximum allowed between batches and at least 2 batches required within a valid sequence. The summarized data presented are the mean proportion of fish performing periodic descents across spawning grounds (Prop_mean), and the minimum (Min_Prop) and maximum proportion (Max_Prop) found at the spawning grounds. Bold font; the method chosen and presented in the main MS.

| *Sex* | *PD_Method* | *Prop_mean* | *Min_Prop* | *Max_Prop* |
| --- | --- | --- | --- | --- |
| **Female** | **50_6_2** | **0.42** | **0.21** | **0.50** |
| Female | 50_6_3 | 0.25 | 0.08 | 0.38 |
| Female | 50_10_2 | 0.44 | 0.21 | 0.56 |
| Female | 50_10_3 | 0.29 | 0.08 | 0.45 |
| Female | 100_6_2 | 0.06 | 0.00 | 0.12 |
| Female | 100_6_3 | 0.03 | 0.00 | 0.08 |
| Female | 100_10_2 | 0.10 | 0.00 | 0.27 |
| Female | 100_10_3 | 0.03 | 0.00 | 0.08 |
| **Male** | **50_6_2** | **0.06** | **0.00** | **0.19** |
| Male | 50_6_3 | 0.00 | 0.00 | 0.03 |
| Male | 50_10_2 | 0.08 | 0.00 | 0.25 |
| Male | 50_10_3 | 0.03 | 0.00 | 0.12 |
| Male | 100_6_2 | 0.01 | 0.00 | 0.06 |
| Male | 100_6_3 | 0.00 | 0.00 | 0.00 |
| Male | 100_10_2 | 0.02 | 0.00 | 0.12 |
| Male | 100_10_3 | 0.00 | 0.00 | 0.00 |
